# Diagnostic efficiency of metagenomic next-generation sequencing for suspected spinal tuberculosis in China: A multicenter prospective study

**DOI:** 10.3389/fmicb.2022.1018938

**Published:** 2022-12-07

**Authors:** Yuan Li, Xiao-wei Yao, Liang Tang, Wei-jie Dong, Ting-long Lan, Jun Fan, Feng-sheng Liu, Shi-bing Qin

**Affiliations:** ^1^Department of Orthopedics, Beijing Chest Hospital, Capital Medical University, Beijing, China; ^2^Department of Orthopedics, Hebei Chest Hospital, Shijiazhuang, China; ^3^Department of Orthopedics, Tianjin Haihe Hospital, Tianjin, China

**Keywords:** diagnosis, spine, tuberculosis, metagenomic next-generation sequencing, infection

## Abstract

**Background:**

The pathogens of suspected spinal tuberculosis (TB) include TB and non-TB bacteria. A rapid and effective diagnostic method that can detect TB and non-TB pathogens simultaneously remains lacking. Here, we used metagenomic next-generation sequencing (mNGS) to detect the pathogens in patients with suspected spinal TB.

**Methods:**

The enrolled patients with suspected spinal TB were regrouped three times into patients with spinal infection and controls, patients with spinal TB and controls, and patients with non-TB spinal infection and controls. We tested the three groups separately by using mNGS and conventional detection methods.

**Results:**

Ultimately, 100 patients were included in this study. Pathogens were detected in 82 patients. Among the 82 patients, 37 had TB and 45 were infected with other bacteria. In patients with spinal infection, the sensitivity of the mNGS assay was higher than that of culture and pathological examination (*p* < 0.001, *p* < 0.001). The specificity of the mNGS assay was not statistically different from that of culture and pathological examination (*p* = 1.000, *p* = 1.000). In patients with spinal TB, no statistical difference was found between the sensitivity of the mNGS assay and that of Xpert and T-SPOT.TB (*p* = 1.000, *p* = 0.430). The sensitivity of the mNGS assay was higher than that of MGIT 960 culture and pathological examination (*p* < 0.001, *p* = 0.006). The specificities of the mNGS assay, Xpert, MGIT 960 culture, and pathological examination were all 100%. The specificity of T-SPOT.TB (78.3%) was lower than that of the mNGS assay (100%; *p* < 0.001). In patients with non-TB spinal infection, the sensitivity of the mNGS assay was higher than that of bacterial culture and pathological examination (*p* < 0.001, *p* < 0.001). The specificity of the mNGS assay was not statistically different from that of bacterial culture and pathological examination (*p* = 1.000, *p* = 1.000).

**Conclusion:**

Data presented here demonstrated that mNGS can detect TB and non-TB bacteria simultaneously, with high sensitivity, specificity and short detection time. Compared with conventional detection methods, mNGS is a more rapid and effective diagnostic tool for suspected spinal TB.

## Introduction

Tuberculosis (TB) is the leading cause of death from a single infectious agent worldwide and remains one of the top 10 causes of death. According to the 2021 global TB report, approximately 5.8 million people fell ill with TB in 2020, and 1.5 million people died of TB globally (Word Health Organization, 2021). Osteoarticular TB is one of the common types of extrapulmonary TB, which currently accounts for 15 ~ 20% of TB case in Asia, and spinal TB accounts for approximately half of bone TB cases (Pigrau-Serrallach and Rodríguez-Pardo, 2013). In recent years, the proportion of non-TB infections in patients with suspected spinal TB has increased gradually (Tsantes et al., 2020). Although non-TB spinal infection and spinal TB have similar clinical symptoms and imaging findings, their treatment is contradictory (Kafle et al., 2022). Thus, their differential diagnosis is required. The misdiagnosis and delayed treatment of suspected spinal TB often lead to death or disability. Therefore, making a rapid and accurate diagnosis becomes the key to controlling these two diseases.

Conventional detection methods, such as culture, targeted nucleic acid amplification tests, and immunological assays, can be challenging due to the wide variety of pathogens that cause clinically indistinguishable diseases. Bacterial culture has been considered as the gold standard for the diagnosis of infectious diseases. However, bacterial culture cannot detect many different types of bacteria simultaneously; for example, BACTEC MGIT 960 (MGIT 960) culture can only detect TB and not other bacteria (Ma et al., 2020; Tsang et al., 2020). Meanwhile, the administration of antimicrobial drugs before culture reduces organism recovery rates. In addition, bacterial culture is a time-consuming method, i.e., the general bacterial culture takes a few days, and TB bacterial culture takes 2 months (Stangenberg et al., 2021; Mingora et al., 2022). Conventional PCR-based tests are targeted methods, and thus cannot be used effectively without some prior knowledge regarding the identity of the pathogen in question. Although Xpert MTB/RIF (Xpert) has good TB detection ability, it cannot detect other bacteria (Ma J. et al., 2022; Wilson et al., 2014). Immunological tests, such as T-SPOT.TB, have poor specificity for TB infections and cannot be used as the main basis for diagnosis; moreover, they reflect previous TB infections (Ryang and Akbar, 2020; Berrocal-Almanza et al., 2022). Therefore, a rapid, accurate and extensive method for the detection of pathogenic microorganisms is the key to solving the above problem.

Metagenomic next-generation sequencing (mNGS) is an unbiased approach to the detection of pathogens. It can overcome the limitations of current diagnostic tests, thus allowing for hypothesis-free, culture-independent pathogen detection directly from clinical specimens. It can cover almost all clinical pathogens that range from viruses to bacteria, fungi, and parasites, and has a short detection time (Gu et al., 2019). In particular, when conventional testing methods cannot provide information about pathogens, mNGS can provide a timely and valuable reference for clinicians in most cases. Previous studies have shown the promises of mNGS as a diagnostic tool for infectious diseases (Simner et al., 2018).

However, only a few reports on the diagnostic efficiency of mNGS for suspected spinal TB exist; furthermore, existing studies have shortcomings, such as small sample sizes and the lack of controls, and are mostly retrospective (Ruppé et al., 2017; Zhao et al., 2020; Ma C. et al., 2022). Therefore, the diagnostic ability of mNGS for suspected spinal TB has not been accurately evaluated. This situation limits the application of mNGS in the clinical diagnosis of suspected spinal TB. Thus, a multicenter prospective study with a large sample size and control group was designed to evaluate systematically the efficacy of mNGS in the diagnosis of suspected spinal TB and to guide its clinical application.

## Materials and methods

### Patient enrollment

From January 2021 to December 2021, patients with suspected spinal TB were prospectively enrolled in the Orthopedics Department of three TB-specialized hospitals: Beijing Chest Hospital, Hebei Chest Hospital, and Tianjin Haihe Hospital. The patients enrolled in the study had clinical manifestations suggestive of suspected spinal TB. These manifestations included (i) persistent back pain lasted for at least 3 weeks, (ii) low fever (< 38°C), (iii) elevated erythrocyte sedimentation rate (male > 15 mm/h, female > 20 mm/h), and (iv) spinal magnetic resonance imaging abnormalities.

### Sample collection and processing

Specimens were collected *via* surgery or CT-guided puncture. Specimens included granulation tissue and pus. Blood specimens were obtained when all patients were enrolled in this study. The samples then were sent to the laboratory for further processing. Granulation tissue samples were cut into small pieces on a disposable Petri dish support by using a scalpel. Each granulation tissue sample was weighed, and then added with phosphate-buffered saline at the rate of 1 g/ml. The mixture was homogenized with a FastPrep-24 instrument (MP Biomedicals Europe) for 100 s at 6 m/s by using MP Bio FASTPREP-24. During homogenization, the tube containing the mixture was removed from the FastPrep-24 instrument every 20 s and cooled on ice for 30 s. Pus samples or the mixtures of homogenized granulation tissue (a 600 μl volume of each) from all patients were each mixed with 1 g of 0.5 mm diameter glass beads and then placed on a vortex mixer for 30 min at 3,000 rpm.

### Conventional testing

Conventional tests included Xpert assay, pathological examination, MGIT 960 culture, and T-SPOT.TB test. The experimental procedure was consistent with our previous studies (Li et al., 2018). Bacterial culture examination: Specimens were tested by using aerobic and anaerobic bacterial cultures. Briefly, abscess specimens were plated and incubated for up to 5 days on 5% sheep blood and MacConkey agar for aerobic culture and on 5% sheep blood agar for anaerobic culture. Bacterial identification was performed with VITEK 2 Compact system (Bio Mérieux, France). Operation was conducted in accordance with the manufacturer’s instructions.

### mNGS

DNA was extracted from 300 μl of each pretreated sample by using a TIANamp Micro DNA Kit (Tiangen Biotech, Beijing, China) in accordance with the manufacturer’s instructions. Purified DNA was fragmented into 200–300 bp segments by using ultrasound followed by end-repair, ligation with multiplex barcode adapters, and PCR amplification to complete the construction of DNA libraries. After the molarities of DNA libraries were estimated by using indexing PCR, the DNA concentrations were determined *via* the DNA Qubit Assay (Thermo Fisher). Meanwhile, DNA quality was evaluated electrophoretically by using an Agilent 2,100 system (Agilent Technologies, Santa Clara, CA, United States). Up to 20 qualified DNA libraries were pooled, and then pooled libraries were subjected to DNA sequencing analysis by using the MGISEQ-2000 platform (MGI Tech Co., Shenzhen, China).

### Bioinformatics analysis

Low-quality sequences and adaptor sequences were first removed to generate clean reads. Subsequently, sequences mapped to the human reference genome (hg19) were subtracted from the clean reads by using Burrows–Wheeler Alignment software (version 0.7.10). Nonhuman sequence reads from each sample were submitted to the Genome Sequence Archive of the Beijing Institute of Genomics, Chinese Academy of Sciences under the accession number PRJCA000880. Additionally, the remaining data were further mapped against the RefSeq Microbial Genome Database of viruses, bacteria, fungi, and parasites by using Burrows–Wheeler Alignment software (version 0.7.10). RefSeq Microbial Genome Database was created and maintained by the National Center of Biotechnology Information. RefSeq analysis yielded 1,798 whole-genome sequences matching the DNA of viral taxa, 6,350 bacterial genomes or scaffolds, 1,064 pathogenic fungi of human infections, and 234 parasites associated with human diseases (Wang et al., 2019). Reporting criteria for infectious pathogens identified by using mNGS included: (i) > 30% relative abundance at the genus level in bacteria or fungi; (ii) at least three unique reads from a single viral, bacterial, or fungal species; and (iii) at least one unique read matching M. TB complex species (Wang et al., 2019). If more than one pathogen was detected, the species present with the greatest relative abundance yielding the highest number of unique reads was deemed as the probable species associated with osteoarticular infection in that patient.

### Patient categories

On the basis of the composite reference standard (CRS), the patients were categorized into three groups: (1) cases with spinal TB infection (including A: cases positive for mycobacterial culture, B: cases with the pathological result of TB and good response to anti-TB therapy, C: cases with the Xpert result of TB and good response to anti-TB therapy); (2) non-TB spinal infection cases (including A: cases with positive bacterial culture, B: cases with the pathological result of infection and good response to anti-infection therapy, and C: cases with the pathological result of inflammation and good response to anti-infection therapy); and (3) non-infection spinal diseases cases (negative results for spinal TB infection and non-TB spinal infection test, and patient improved without receiving anti-TB and anti-infection therapy). Pathological result of TB includes typical tuberculous granuloma or positive acid-fast staining. Pathological result of infection includes presence of white blood cells and pus cells. A good outcome was defined as follows: (1) resolution of clinical symptoms due to infection, (2) improvement of osteoarticular function, and (3) improvement of inflammation, as indicated by inflammatory biomarkers and radiological features (Wieland et al., 2012).

### Statistical analysis

The demographic and clinical data of the study subjects were collected by using case report forms. The data included gender, age, comorbidities, clinical symptoms, laboratory results, radiological features, and treatment regimens. All data were entered through double manual data entry with the EpiData Entry program, version 3.1 (EpiData Association, Odense, Denmark). Statistical analysis was performed by using SPSS software, version 20.0 (IBM SPSS, Chicago, IL, United States). Chi-square test and Fisher’s exact test were used for categorical variables, whereas *t*-test or Mann–Whitney *U*-test was used for continuous variables, as appropriate. A two-sided value of *p* < 0.05 was considered statistically significant.

## Results

### Study patients

A total of 114 consecutive patients with suspected spinal TB were prospectively enrolled. Subsequently, specimens were obtained from 100 of the 114 patients through surgery or CT-guided puncture. Ultimately, 100 patients were included in this study. In accordance with the CRS, 38 patients were diagnosed with spinal TB, 53 patients were diagnosed with non-TB spinal infection, and nine patients were diagnosed with non-infectious spinal diseases. Table 1 shows the demographic characteristics of the studied patients in different categories.

**Table 1:** Clinical characteristics of the studied patients.

Clinical characteristics	Spinal TB infection	Non-TB spinal infection	Non-infection spinal diseases
	(*n* = 38)	(*n* = 53)	(*n* = 9)
Age, years (mean ± SD)	44.1 ± 15.9	51.4 ± 16.5	49.00 ± 17.2
Female, *n* (%)	20 (52.6%)	23 (43.4%)	5 (55.6%)
Site of spine lesion, *n* (%)			
Lumbar vertebra	19 (50.0%)	36 (67.9%)	4 (44.4%)
Thoracic vertebra	16 (42.1%)	13 (24.5%)	5 (55.6%)
Cervical vertebra	3 (7.9%)	4 (7.6%)	0 (0.0%)
Clinical symptoms			
Fever	13 (34.2%)	31 (58.5%)	0 (0.0%)
Pain	28 (73.7%)	43 (81.1%)	7 (77.8%)
Complications			
Pulmonary tuberculosis	7 (18.4%)	0 (0.0%)	0 (0.0%)
Diabetes	8 (21.1%)	17 (32.1%)	1 (11.1%)
Autoimmune disease	5 (13.2%)	9 (17.0%)	0 (0.0%)

N: number of patients, TB: tuberculosis

### mNGS assay

The detection time of mNGS ranged from 15.0 to 20.5 h, with an average of 17.7 ± 1.7 h. The numbers of sequence reads ranged from 3.2 × 10^6^ to 6.3 × 10^7^ reads, with an average of (2.7 ± 2.0) × 10^7^ reads per specimen. The sequencing depth of mNGS for pathogens ranges from 1.0 × to 6.5 ×, with an average of (3.4 ± 1.7) × per specimen (Supplementary Table S1). The bacteria detected by mNGS and culture in this study were consistent.

### Pathogen composition

**Figure 1:**
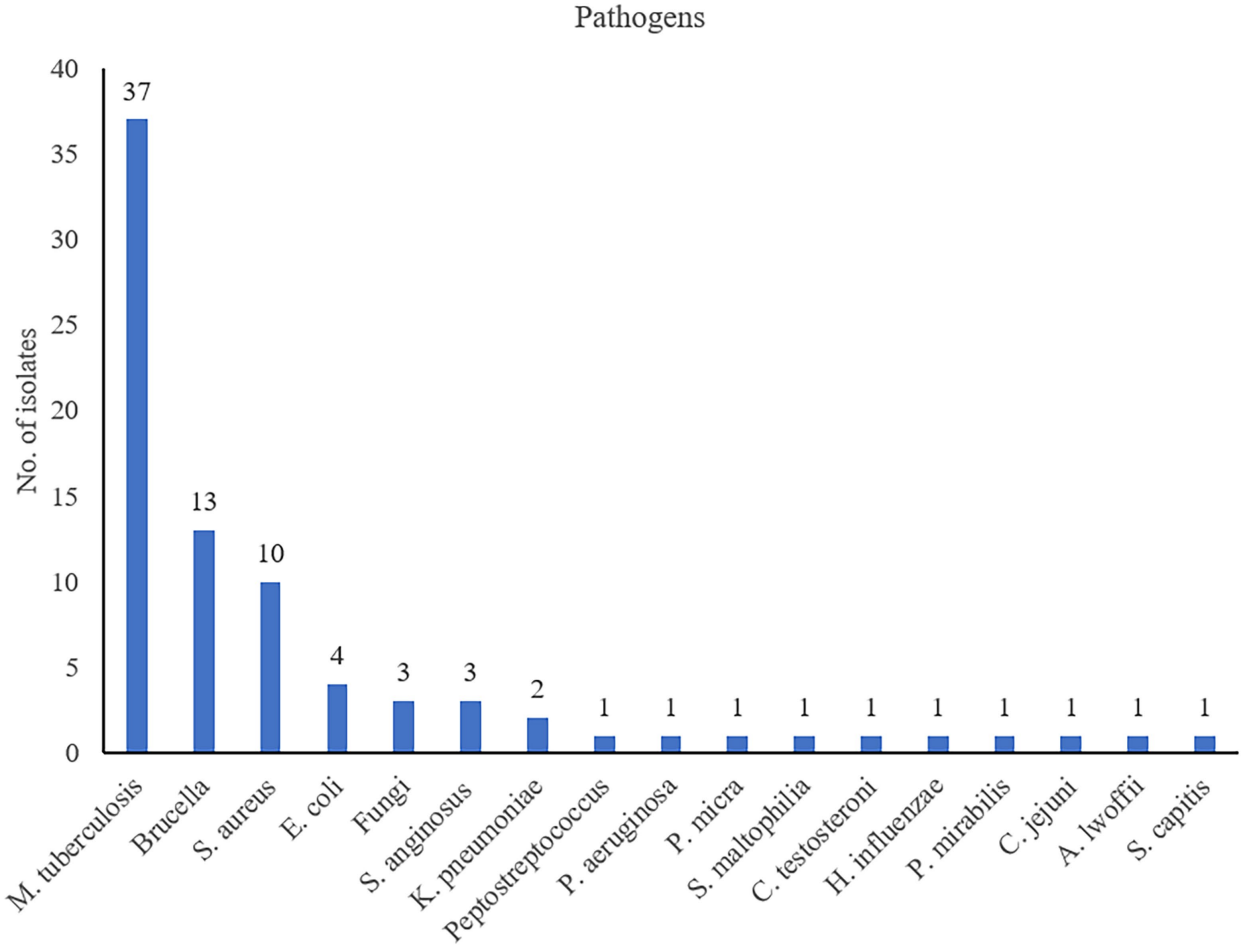
Composition of pathogens detected in 82 patients with spinal infection.

In this study, pathogens were detected in 82 patients with spinal infection by using mNGS, MGIT 960 culture, bacterial culture, and Xpert. Among the 82 patients, 37 had TB and 45 were infected with other bacteria. *Brucella*, *Staphylococcus aureus*, *Escherichia coli*, fungi, *Streptococcus anginosus*, and *Klebsiella pneumoniae* accounted for 28.9% (13/45), 22.2% (10/45), 8.9% (4/45), 6.7% (3/45), 6.7% (3/45), and 4.4% (2/45) of the 45 non-TB bacteria. In addition, 10 of the 45 non-tuberculous bacteria were detected only once. The 3 fungi included 1 *Candida albicans*, 1 *Candida glabrata* and 1 *Candida parapsilosis*. Figure 1 shows the composition of pathogens detected in 82 patients with spinal infection.

### Performance of mNGS and conventional detection methods in patients with spinal infection

In accordance with the CRS, patients with spinal infection included patients with spinal TB and patients with non-TB spinal infection, and patients with non-infection spinal diseases were used as the control group. In all patients with spinal infection, the sensitivities of the mNGS assay, culture (including MGIT 960 and bacterial cultures), and pathological examination were 89.0% (81/91), 28.1% (25/89), 42.9% (30/70), respectively. The sensitivity of the mNGS assay was higher than that of culture and pathological examination. Moreover, the sensitivity of the mNGS assay was statistically different from that of culture and pathological examination (*p* < 0.001, *p* < 0.001). In all patients with spinal infection, the specificities of the mNGS assay, culture (including MGIT 960 and bacterial cultures), and pathological examination were 88.9% (8/9), 100.0% (9/9), and 100.0% (9/9), respectively. The specificity of the mNGS assay was not statistically different from that of culture and pathological examination (*p* = 1.000, *p* = 1.000). Table 2 shows the performances of mNGS and conventional detection methods in patients with spinal infection.

**Table 2:** Performance of mNGS and conventional detection methods in patients with spinal infection.

Methods	Sensitivity	Specificity	PPV	NPV	*p* value	*p* value
	(%, *N*, 95% CI)	(%, *N*, 95% CI)	(%, *N*, 95% CI)	(%, *N*, 95% CI)	(sensitivity)	(specificity)
mNGS	89.0% (81/91)	88.9% (8/9)	98.8% (81/82)	44.4% (8/18)	–	–
	(83–96)	(52–100)	(96–101)	(22–69)		
Culture	28.1% (25/89)	100.0% (6/6)	100.0% (25/25)	8.6% (6/70)	*χ*^2^ = 68.997	*p* = 1.000^a^
	(19–38)	(54–100)	(86–100)	(2–15)	*p* < 0.001^a^	
Pathological examination	42.9% (30/70)	100.0% (9/9)	100.0% (30/30)	18.4% (9/49)	*χ*^2^ = 39.636	*p* = 1.000^b^
	(31–55)	(66–100)	(88–100)	(9–32)	*p* < 0.001^b^	

N: number of patients, PPV: positive predictive value, NPV: negative predictive value, CI: confidence interval, a: mNGS vs. culture.

b : mNGS vs. Pathological examination.

### Performance of mNGS and conventional detection methods in patients with spinal TB

The enrolled patients were divided into two groups in accordance with the CRS. One group comprised patients with spinal TB, and the other group, which included patients with non-TB spinal infection and non-infection spinal diseases, served as the control. In patients with spinal TB, the sensitivities of the mNGS assay, Xpert, MGIT 960 culture, pathological examination, and T-SPOT.TB. were 94.7% (36/38), 94.6% (35/37), 45.9% (17/37), 67.9% (19/28), and 89.2% (33/37) respectively. No statistical difference was found between the sensitivity of the mNGS assay and that of Xpert and T-SPOT.TB (*p* = 1.000, *p* = 0.430). Compared with that of MGIT 960 culture and pathological examination, the sensitivity of the mNGS assay was higher and was statistically different (*p* < 0.001, *p* = 0.006). In patients with spinal TB, the specificities of the mNGS assay, Xpert, MGIT 960 culture, and pathological examination were all 100%. In patients with spinal TB, the specificity of T-SPOT.TB (78.3%) was lower than that of the mNGS assay (100.0%), and the difference between the specificities of these assays were statistically significant (*p* < 0.001). Table 3 shows the performances of mNGS and conventional detection methods in patients with spinal TB.

**Table 3:** Performance of mNGS and conventional detection methods in patients with spinal TB.

Methods	Sensitivity	Specificity	PPV	NPV	*p* value	*p* value
	(%, *N*, 95% CI)	(%, *N*, 95% CI)	(%, *N*, 95% CI)	(%, *N*, 95% CI)	(sensitivity)	(specificity)
mNGS	94.7% (36/38)	100.0% (62/62)	100.0% (36/36)	96.9% (62/64)	–	–
	(82–99)	(94–100)	(90–100)	(89–100)		
Xpert	94.6% (35/37)	100.0% (56/56)	100.0% (35/35)	96.6% (56/58)	*p* = 1.000^a^	–
	(82–99)	(94–100)	(90–100)	(88–100)		
MGIT 960 culture	45.9% (17/37)	100.0% (41/41)	100.0% (17/17)	67.2% (41/61)	*χ*^2^ = 68.997	–
	(30–63)	(91–100)	(80–100)	(55–79)	*p* < 0.001^b^	
Pathological examination	67.9% (19/28)	100.0% (51/51)	100.0% (19/19)	85.0% (51/60)	*p* = 0.006^c^	–
	(48–84)	(93–100)	(82–100)	(76–94)		
T-SPOT.TB	89.2% (33/37)	78.3% (47/60)	71.7% (33/46)	92.2% (47/51)	*p* = 0.430^d^	*χ*^2^ = 15.035
	(75–97)	(68–89)	(57–84)	(85–100)		*p* < 0.001^d^

N: number of patients, PPV: positive predictive value, NPV: negative predictive value, CI: confidence interval, ^a^mNGS vs. Xpert, ^b^mNGS vs. MGIT 960 culture, ^c^mNGS vs. pathological examination, ^d^mNGS vs. T-SPOT.TB.

### Performance of mNGS and conventional detection methods in patients with non-TB spinal infection

The enrolled patients were divided into two groups on the basis of the CRS. One group comprised patients with non-TB spinal infections, and the other group, which served as the control, constituted patients with spinal TB and non-infection spinal diseases. The sensitivities of the mNGS assay, bacterial culture, and pathological examination in patients with non-TB spinal infection were 84.9% (45/53), 15.4% (8/52), 26.2% (11/42), respectively. Compared with that of bacterial culture and pathological examination, the sensitivity of the mNGS assay was higher and statistically different (*p* < 0.001, *p* < 0.001). The specificities of the mNGS assay, bacterial culture, and pathological examination in patients with non-TB spinal infection were 97.9% (46/47), 100.0% (43/43), and 100.0% (37/37), respectively. The specificity of the mNGS assay was not statistically different from that of bacterial culture and pathological examination (*p* = 1.000, *p* = 1.000). Table 4 shows the performances of the mNGS and conventional detection methods in patients with non-TB spinal infection.

**Table 4:** Performance of mNGS and conventional detection methods in patients with non-TB spinal infection.

Methods	Sensitivity	Specificity	PPV	NPV	*p* value	*p* value
	(%, *N*, 95% CI)	(%, *N*, 95% CI)	(%, *N*, 95% CI)	(%, *N*, 95% CI)	(sensitivity)	(specificity)
mNGS	84.9% (45/53)	97.9% (46/47)	97.8% (45/46)	85.2% (46/54)	-	-
	(75–95)	(88–100)	(88–100)	(75–95)		
Bacterial culture	15.4% (8/52)	100.0% (43/43)	100.0% (8/8)	49.4% (43/87)	*χ*^2^ = 50.748	*p* = 1.000^a^
	(5–26)	(91–100)	(63–100)	(39–60)	*p* < 0.001^a^	
Pathological examination	26.2% (11/42)	100.0% (37/37)	100.0% (11/11)	54.4% (37/68)	*χ*^2^ = 33.381.	–
	(14–42)	(90–100)	(72–100)	(42–67)	*p* < 0.001^b^	

N: number of patients, PPV: positive predictive value, NPV: negative predictive value, CI: confidence interval, ^a^mNGS vs. bacterial culture, ^b^mNGS vs. pathological examination

## Discussion

Infectious diseases remain the leading causes of morbidity and mortality in all patient populations worldwide. They are accompanied by a mortality rate of approximately 15% (Michiels and Jäger, 2017). Accurate diagnosis can be challenging due to the wide variety of pathogens that cause clinically indistinguishable diseases. The global TB epidemic remains serious. Spinal TB is a special spinal infectious disease that accounts for < 25% of spinal infectious diseases in Southern China (Yee et al., 2010). In recent years, the proportion of non-TB spinal infections in patients with suspected spinal TB has gradually increased in TB-specialized hospitals in China. In this study, non-TB spinal infection accounted for 53% of the patients with suspected spinal TB, whereas spinal TB accounted for only 38% of the patients. The data showed that the proportion of patients with spinal TB in patients with suspected spinal TB is significantly lower than before, whereas the proportion of spinal infection is significantly higher. This trend is similar to the results reported in the literature (Tsantes et al., 2020; Word Health Organization, 2021).

In this study, *Brucella* and *S. aureus* accounted for a high proportion of the non-TB spinal infection bacteria and were present at considerably higher proportions than other bacteria. *S. aureus* is a common bone infection pathogen. The high proportion of *Brucella* is mainly due to the close location of the three hospitals to pastoral areas in northern China. At the same time, given that *Brucella* infection has similar symptoms as TB infection, the patients went to TB-specialized hospitals for treatment. Opportunistic pathogens also account for a certain proportion of cases with non-TB bacterial spinal infections (Gupta et al., 2022; Mehkri et al., 2022).

The accurate and rapid differential diagnosis of the two diseases poses a new challenge to the clinicians. Given that conventional detection methods cannot meet the needs of clinical diagnosis, so new technologies need to be introduced to solve the existing problems. mNGS is an unbiased approach for pathogen detection that allows for universal pathogen detection regardless of the type of pathogen (viruses, bacteria, fungi, and parasites), and mNGS can even be applied for novel organism discovery (Gu et al., 2019). Therefore, mNGS is a powerful tool for differential diagnosis in patients with suspected spinal TB. Some studies have shown that mNGS performs well in the diagnosis of orthopedic infectious diseases (Ruppé et al., 2017; Ma C. et al., 2022). However, only a few reports on the ability of mNGS for the differential diagnosis ability of suspected spinal TB exist (Huang et al., 2019, 2020; Zhao et al., 2020). Therefore, a multicenter prospective study was conducted to evaluate the diagnostic performance of mNGS in patients with suspected spinal TB.

In this study, the sensitivity of mNGS (89.0%) was significantly higher than that of culture (28.1%) and pathological examination (42.9%) in patients with spinal infection. These results demonstrated the higher sensitivity of mNGS than that of conventional detection methods. However, mNGS had a lower specificity (88.9%) than culture (100.0%) and pathology (100.0%) because it detected bacteria in one uninfected patient. This result is similar to previously reported findings (Zhou et al., 2019; Ma C. et al., 2022). In this study, mNGS detected *Porphyromonas gingivalis* in a specimen from an uninfected patient. This situation indicated the possibility of false-positives in mNGS results. *P. gingivalis* was likely detected in the uninfected patient because it is a bacterium that commonly colonizes the oral cavity. Therefore, mNGS should be combined with other clinical tests to enable comprehensive judgment. False-positive results in mNGS testing may be related to contamination, unbiased nucleic acid amplification, and human-colonizing bacteria.

In this study, mNGS and Xpert showed similar sensitivity (94.7% vs. 94.6%) and the same specificity (100.0%) without statistically different sensitivities (*p* = 1.000) for spinal TB. This result suggested that mNGS and Xpert have the comparable abilities for the diagnosis of spinal TB. The sensitivity of T-SPOT.TB was not statistically significantly different from that of mNGS and Xpert (89.2% vs. 94.7%, 94.6%). However, T-SPOT.TB had significantly lower specificity than mNGS and Xpert (78.3% vs. 100.0%, 100.0%). Therefore, the diagnostic capability of T-SPOT.TB was lower than that of mNGS and Xpert. This finding is similar to the reported results (Zhao et al., 2020; Chen et al., 2022). The poor specificity of T-SPOT.TB is mainly due to the high latent infection rate of mycobacterium TB in China (Word Health Organization, 2021). MGIT 960 culture and pathological examination had significantly lower sensitivity than mNGS (45.9%, 67.9% vs. 94.7%). However, the specificity of these methods was consistent with that of mNGS (100.0%). This result suggested that the ability of these methods to diagnose spinal TB is lower than that of mNGS.

In patients with non-TB spinal infection, the sensitivity of mNGS was significantly higher than that of bacterial culture and pathological examination (84.9% vs. 15.4%, 26.2%). The specificity of mNGS was 97.9%, and although mNGS had a false-positive result for one case, no statistical difference was found among the specificities of mNGS, bacterial culture, and pathological examination. This result indicated that mNGS has a good diagnostic ability in the detection of non-TB spinal infection and is similar to previously reported findings (Miao et al., 2018; Huang et al., 2020). Given the existence of false-positive test results, the results of mNGS junction tests should be comprehensively analyzed in combination with clinical conditions.

The results of this study show that mNGS has higher sensitivity and specificity than conventional detection methods in the diagnosis of spinal TB and non-TB spinal disease infection, except for Xpert. Therefore, mNGS is a powerful diagnostic tool for patients with suspected spinal TB and avoids missed diagnosis and misdiagnosis. However, conventional methods also have their own advantages over mNGS; for example, although Xpert has the same ability as mNGS for the diagnosis of spinal TB, it can detect rifampicin-resistant gene mutations and provide guidance for TB treatment plans (Yu et al., 2021). In addition, MGIT 960 and bacterial cultures can be used to conduct drug sensitivity tests on bacteria (Zhou et al., 2019; Chen et al., 2022), and the results of drug sensitivity test have important guiding significance for the treatment of clinical spinal infections. Currently, mNGS provides limited information on the drug sensitivity of the detected bacteria, and its ability to detect drug-resistant mutations needs further study.

In this study, mNGS did not detect bacteria in ten patients with spinal infection and detected bacteria in an uninfected patient. Currently, mNGS also has some problems in the diagnosis of infectious diseases. These problems are related to the sequencing principle of mNGS. Given that mNGS indiscriminately detects all nucleic acid molecules in specimens, including pathogenic bacteria, colonized bacteria, and exogenous nucleic acid molecules previously integrated into the human body, pathogens need to be distinguished from other bacteria. At the same time, the possibility of contamination, including contamination from specimens, reagents, and operating procedures, exists. mNGS provides a massive amount of data, 90% of which is on human nucleic acids, and requires information analysis to exclude interfering factors and identify pathogenic bacteria (Simner et al., 2018; Xiao et al., 2022). For the samples with low pathogen load, the sequencing depth of mNGS should be increased to improve the detection rate of bacteria, but this will lead to the increase of sequencing cost and sequencing time. Therefore, it is necessary to balance the relationship between the sequencing cost, sequencing depth, and sequencing time of mNGS from the aspects of sample processing, detection process, and bioinformatics analysis.

This study had several limitations. First, three TB-specialized hospitals were selected as research units. Therefore, the representation of pathogen com position is limited to a certain proportion. Second, RNA sequencing was not carried out because RNA is unstable and easily degraded. Therefore, pathogenic microorganism with RNA genomes could not be detected. Third, the sensitivity of nonhuman DNA detection was removed because human DNA contamination was not depleted during sample DNA purification.

In summary, mNGS is a rapid and effective diagnostic tool for patients with suspected spinal TB. In contrast to conventional detection method, mNGS can detect tuberculous and non-TB bacteria infection simultaneously, thus avoiding missed diagnosis and misdiagnosis. mNGS also has high sensitivity, specificity and short detection time. Nevertheless, mNGS also has some shortcomings. Thus, further research is needed.

## Data availability statement

The original contributions presented in the study are included in the article/Supplementary material, further inquiries can be directed to the corresponding authors.

## Ethics statement

The studies involving human participants were reviewed and approved by ethics committee of Hebei Chest Hospital. The patients/participants provided their written informed consent to participate in this study.

## Author contributions

S-bQ, F-sL, and YL designed the study. YL, LT, W-jD, JF, T-lL, and X-wY participated in data collection. YL, X-wY, S-bQ, and F-sL participated in data analysis. YL, X-wY, LT, W-jD, JF, and T-lL wrote the manuscript. Author ranking based on contributions to articles. All authors contributed to the article and approved the submitted version.

## Funding

This study was funded by the National Science and Technology Major Project (grant number 2017ZX09304009-004; http://www.nmp.gov.cn/). The funders had no role in the study design, data collection and analysis, decision to publish, or preparation of the manuscript.

## Conflict of interest

The authors declare that the research was conducted in the absence of any commercial or financial relationships that could be construed as a potential conflict of interest.

## Publisher’s note

All claims expressed in this article are solely those of the authors and do not necessarily represent those of their affiliated organizations, or those of the publisher, the editors and the reviewers. Any product that may be evaluated in this article, or claim that may be made by its manufacturer, is not guaranteed or endorsed by the publisher.
