# Corrigendum: Diagnostic efficiency of metagenomic next-generation sequencing for suspected spinal tuberculosis in China: a multicenter prospective study

**DOI:** 10.3389/fmicb.2023.1198931

**Published:** 2023-04-18

**Authors:** Yuan Li, Xiao-wei Yao, Liang Tang, Wei-jie Dong, Ting-long Lan, Jun Fan, Feng-sheng Liu, Shi-bing Qin

**Affiliations:** ^1^Department of Orthopedics, Beijing Chest Hospital, Capital Medical University, Beijing, China; ^2^Department of Orthopedics, Hebei Chest Hospital, Shijiazhuang, China; ^3^Department of Orthopedics, Tianjin Haihe Hospital, Tianjin, China

**Keywords:** diagnosis, spine, tuberculosis, metagenomic next-generation sequencing, infection

In the published article, there was an error in the numbering of affiliations. Instead of “Wei-jie Dong^2^, Ting-long Lan^2^, Jun Fan^2^, Feng-sheng Liu^3^^*^”, it should be “Wei-jie Dong^1^, Ting-long Lan^1^, Jun Fan^1^, Feng-sheng Liu^2^^*^”.

The authors apologize for this error and state that this does not change the scientific conclusions of the article in any way. The original article has been updated.

